# Measurement of the Aspherical Optical Surfaces with the Improved Phase Retrieval

**DOI:** 10.3390/mi13040549

**Published:** 2022-03-30

**Authors:** Xinxue Ma, Jianli Wang, Bin Wang, Xinyue Liu

**Affiliations:** Changchun Institute of Optics, Fine Mechanics and Physics, CAS, Changchun 130033, China; wangjianli@ciomp.ac.cn (J.W.); wangbin@ciomp.ac.cn (B.W.); liuxinyue@ciomp.ac.cn (X.L.)

**Keywords:** aspherical, metrology, surfaces measurement, phase retrieval

## Abstract

In order to verify the estimated wave-front ability of the phase retrieval, a method utilized in the measurement of the aspherical optical surfaces using the phase retrieval technology is described. This technique is based on the algorithm as a solution for the measurement of the aspherical optical surfaces, whose principle is sampling a number of the given defocus images and obtaining the phase information by solving the wave-front with Fourier optical diffractive theory and mathematics optimization. We set up an experimental arrangement used to measure the aspherical optical surfaces using the improved phase retrieval. In addition, we introduced the method of optical alignment in detail, which is very important for high-precision measurements. We obtained an agreement among the error distributions, the peak value, and the root-mean-square value of a ZYGO interferometer, which demonstrates that the improved phase retrieval method can effectively estimate the wave-front and the aberrations of aspherical optical surfaces.

## 1. Introduction

The free-form optical surface is commonly used as it has better performance and compactness because of the recent advances in optical design and fabrication [1,2,3]. The high-precision free-form optical surface metrology remains difficult because the free-form optical surface has more degrees of freedom for correcting optical aberrations, which means that high-precision free-form optical surface metrology is still a challenge [4,5,6]. Currently, on the market, there are commercial products [7,8,9,10,11] such as Vecco, Taylor-Hobson, and other products that can only analyze the surface accuracy of the aspheric surface, for arbitrary freeform, the original measurement data of the surface shape can be obtained by the company’s Wyko, Talysuf PGI, and other products, however, for the precision analysis of freeform, there is no mature technology and methods. Taylor-Hobson’s Talymap software is used to remove the current shape from the fitting one to obtain the surface shape error, but is affected by the measurement errors, so the evaluation cannot fully realize the freeform of submicron surface shape error, and lack of robustness, even so, the sale price of the software is more than HK$ 1 million. The Japan Panasonic Company claims that they can assess part of the freeform shape error, but its feasibility and standard remain to be determined, and the technology is still a state secret. In academic research, a large number of scholars have proposed different methods, but the accuracy of submicron surface shape and the uncertainty of data processing are still unsolved.

Therefore, in order to solve the problem of free-form optical surface metrology, many metrology methods have been developed [12,13,14], which are roughly divided into contact metrology and non-contact metrology. For high-precision optical surface, contact measurement could easily scratch the surface as there is a certain amount of measurement force in the stylus and the working surface, which increases with a range increase. Under a certain measurement pressure, sharp contact with the workpiece surface will be lost, and affect the authenticity of the measurement results. Non-contact measurement methods are the interference method and scanning method, where scanning methods include the color confocal method, differential confocal method, and so on, but the accuracy of general scanning method is not high, the measurement range is not large, and the scanning speed is slow and this method cannot fulfill high-precision, fast, arbitrary freeform testing. The interferometry includes the sub-aperture splicing technique and computer-generated holography (CGH) technique. Using the sub-aperture stitching technique, the transverse and longitudinal dynamic range of the interferometer can be extended to greatly improve the caliber and relative aperture of the interferometer to measure the optical element, and can greatly improve the spatial resolution of the measurement and reduce the cost. However, the axial shift and inclination of the sub-aperture will greatly reduce the accuracy of the measurement. The main problem of sub-aperture splicing measurements is the error accumulation in the splicing measurement process, so it is important to learn how to eliminate the splicing error, especially the error correction of the aspheric splicing measurement [15,16]. CGH is a diffractive optical element that produces any desired shape wave-front, and can be used as the zero compensator to detect the optical freeform. However, when the surface slope is too large, the marks of CGH, which are used as a zero compensator, will be very dense, and the processing error increases, so the accuracy will decline. For freeform, for each surface testing, a CGH or zero lens is needed, which greatly increases the cost of testing [17,18].

The phase retrieval (PR) method of the non-contact measurement pays more attention to the algorithm design. It mainly depends on the PR algorithm to obtain the final detection results, which is flexible to realize. It can dynamically test optical components and systems and has good application prospects in the fields of optical processing, system assembly and adjustment, active optics, and adaptive optics. PR technology can be used in the field of MEMS or MOEMS systems. For example, in the field of M(O)MES or MEMS based digital micro-mirror devices (DMDs) in the field of low light level electromechanical systems, these elements can replace the space light modulator to control the wave-front (such as generating a specific form of Zernike aberration) and can calibrate the micro mirror deformation control temperature stability of the device. In the manufacturing process of diffractive optical elements, for phase binary optical elements, specific phase structures can be generated in this way before surface microstructure processing and manufacturing [19]. Thus, PR has been favored by experts.

In order to verify the ability of PR in the measurement of the free-form optical surfaces [20,21], many papers have set up a measurement experiment platform with the method of PR [22,23,24,25]. In the early stage, we conducted relevant work on the measurement of small-slope free-form optical surfaces [26], and the main purpose of this paper was to further illustrate and verify the feasibility of our method in the measurement of small-slope aspherical optical surfaces. As we know, the algorithm is the soul of PR, but limited iterative uncertainty and slow convergence speed of the traditional PR algorithm limits the development of PR technology in the measurement of free-form optical surfaces [27,28]. Therefore, we used the improved PR algorithm, which potentially has the advantage of improving the efficiency of phase recovery to solve the limitations of the traditional PR in iterative uncertainty and slow convergence speed [29,30].

## 2. Theory of PR

### The Principle of PR

**Figure 1:**
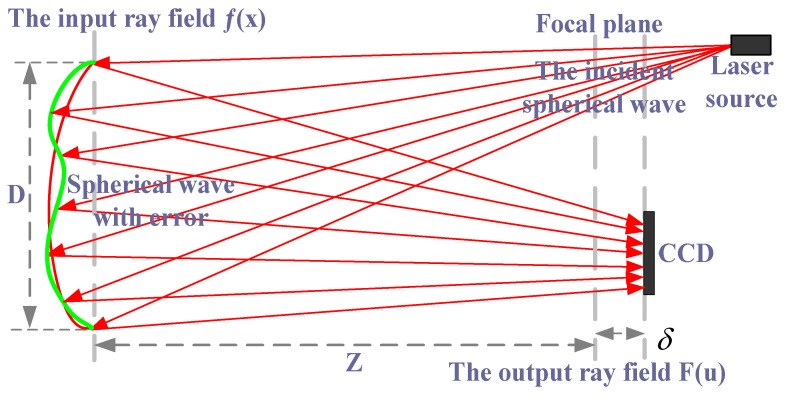
The principle of the PR system [26].

**Figure 2:**
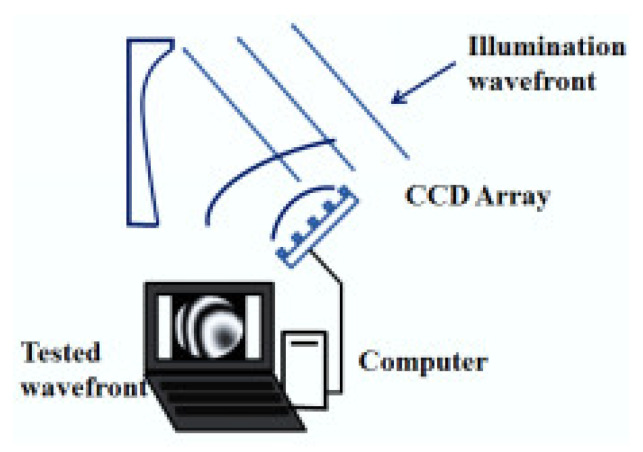
The schematic of optical surface measurement with PR.

The schematic layout of the PR principle is shown in Figure 1. The basic principle of PR is to illuminate the measured mirror by a point light source, obtain the light intensity pattern near the focal plane of the reflected or transmitted beam through CCD, and recover the surface error of the measured mirror through the phase recovery algorithm. The measurement of an optical surface with PR is shown in Figure 2. A known illumination field is reflected off the surface under testing. The resulting field propagates through free space to a plane where the resulting intensity pattern is measured using a detector array such as a charge coupled device (CCD). From this measurement, the PR algorithm computes an estimate of the wave-front [31].

For a linear optical system and a PR system, let f(x) be defined as the generalized pupil function, and its pupil constraint function is |f(x)|. When the defocus of f(x) is δ in the plane, the impulse response function is |F(u)|. For a known optical system, it corresponds to the size and shape of the pupil, and |f(x)| is the prior conditions. ${\left|F(u)\right|}^{2}$ is the image collected by CCD where the defocus is δ. Therefore, the purpose of estimating the wave-front by PR is to obtain $\alpha_{n}$ by the above known quantity. Therefore, formal description of the problem for |f(x)|, $\delta_{1}$, ${\left|F_{1}(u)\right|}^{2}$, $\delta_{2}$, ${\left|F_{2}(u)\right|}^{2}$, …, $\delta_{M}$, ${\left|F_{M}(u)\right|}^{2}$ is known. Image acquisition distance from the focal plane at $\delta_{1}$, $\delta_{2}$, …, $\delta_{M}$ is ${\left|F_{1}(u)\right|}^{2}$, ${\left|F_{2}(u)\right|}^{2}$, …, ${\left|F_{M}(u)\right|}^{2}$, respectively.

In this paper, we will not elucidate the improved PR algorithm in detail; the elaborate process is described in [26].

## 3. The Design of the Experiments

**Figure 3:**
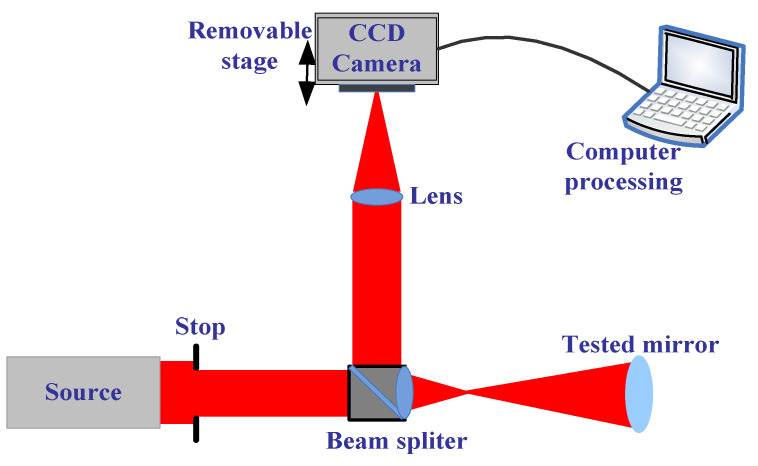
The schematic of the aspherical surface mirror measurement with the improved PR.

Here, we demonstrate the measurement ability in free-form surfaces with the improved phase retrieval discussed in Section 2. In addition, we built the experiment setup with the improved phase retrieval as a method to measure the aspherical optical surfaces. The experimental optical path is shown in Figure 3. Focal length of L3 in the experimental system was 0.12 m, the center wavelength was 632.5 nm, the exit pupil caliber was 0.005 m, and the depth of focus was about 0.73 mm. In the experiment, the defocus amounts we selected were −2.2 mm, −1.7 mm, −1.2 mm, 0 mm, 1.2 mm, 1.7 mm, and 2.2 mm; the PV of the phase of the corresponding defocus was 0λ and 1.6λ. The camera pixel size was 6.45 μm, where each defocused position respectively intercepted a 128 × 128 pixel size of the target region, the exposure time was 20 ms, and the accuracy of removable platform was ±5 μm.

### 3.1. Adjustment of the Optical Path

In order to obtain good measurement results, there are two important keys: one is the algorithms, and the other is how to adjust the optical path accurately, especially in the phase retrieval experiments. Here, we introduce how to adjust the optical path perfectly.

**Figure 4:**
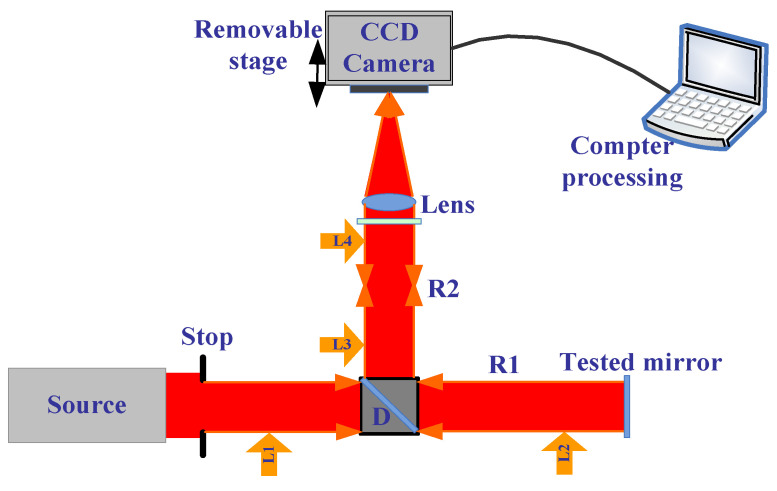
The optical path of testing the plane mirror.

There are four important parts in the experiments shown in Figure 4. The first is how to obtain collimated light from the source. Here, the light from the collimation package (F810FC-635, NA = 0.25, f = 35.41 mm) pre-aligned to collimate the laser beam propagating from the tip of the fiber with diffraction-limited performance at the design wavelength was used as was a small parallel light tube to obtain the spherical wave that was the most uniform and strongest. We used a shear-plate (2.5–5.0 mm beam diameter) to see whether it was collimated.

The second step is how, after the light (called L1) has gone through a Pellicle beam splitter, to make sure that the light with the information of the tested mirror can come back after reflecting (called L2) coincides with L1. If L1 coincides with L2, we also considered the situation that after L2 goes through lens1, it can coincide with L1. Here, we must use iteration methods to make L1 coincide with L2.

The third is how to make sure R2 is coaxial. We used a plan mirror without lens2, so the light passed through L2 and was divided into two parts; we denoted the light in the path of R2 as L3. L3 gets to the plan mirror, and then reflects with information (called L4), adjusts the locations of the plan mirror to make sure two light spots coincide, then puts on lens2, and adjusts the locations of lens2 with the iteration method to make sure that two light spots coincide, then the plan mirror is removed.

The last part is to adjust the location of lens1 and ensure collimated light in R2; we could see from the shear-plate or when we moved the location of the white paper along R2 to check that the spot was the same size, and obtained the right location of lens1.

**Figure 5:**
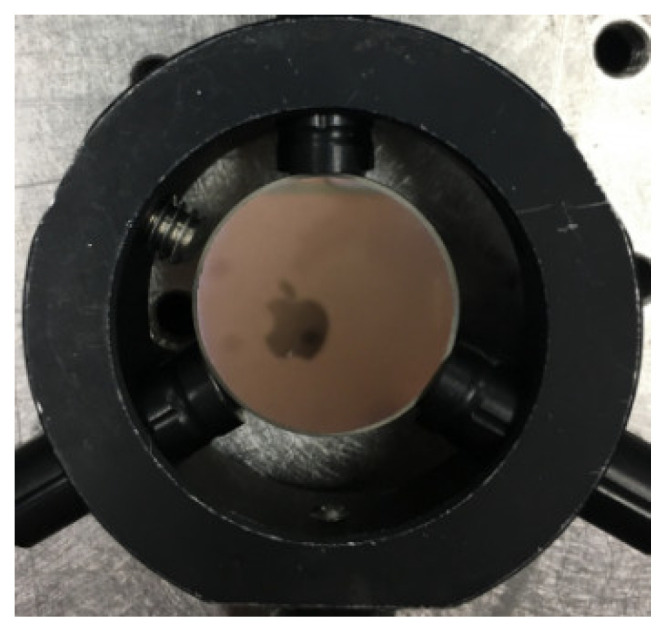
The plan mirror.

**Figure 6:**
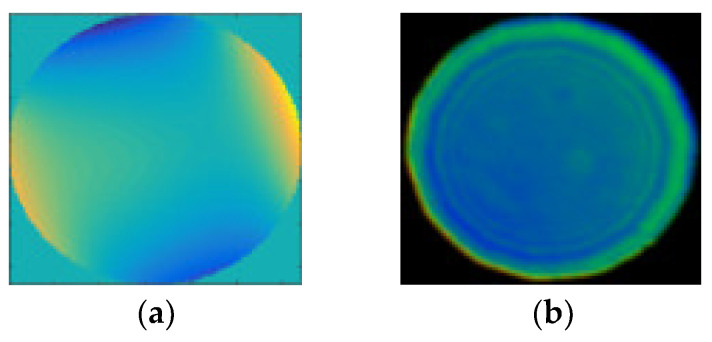
The measurement results with (**a**) PR: root-mean-square (RMS) = 0.0392λ, peak value (PV) = 0.2501λ; (**b**) ZYGO: RMS = 0.023λ, PV = 0.294λ.

In order to obtain accurate results, we must first understand the system aberration. Thus, we measured the plan mirror, as shown in Figure 5. The measurement result with PR is shown in Figure 6a, and the measurement result with ZYGO is shown in Figure 6b. In order to obtain accurate measurements, the aberration of the system itself should be subtracted whenever the aspherical mirror is measured.

### 3.2. The Steps of the Experiments

First: Set up the experimental system, and make sure the light source is collimation.

Second: The collimation light goes through D (has less aberrations), and the light is divided into two parts: one is called R1, and the other can be neglected. In R1, there are two conditions: one is without any lens to measure the plan mirror, and the other is a lens to measure the spherical mirror, the aspherical mirror, and so on.

**Figure 7:**
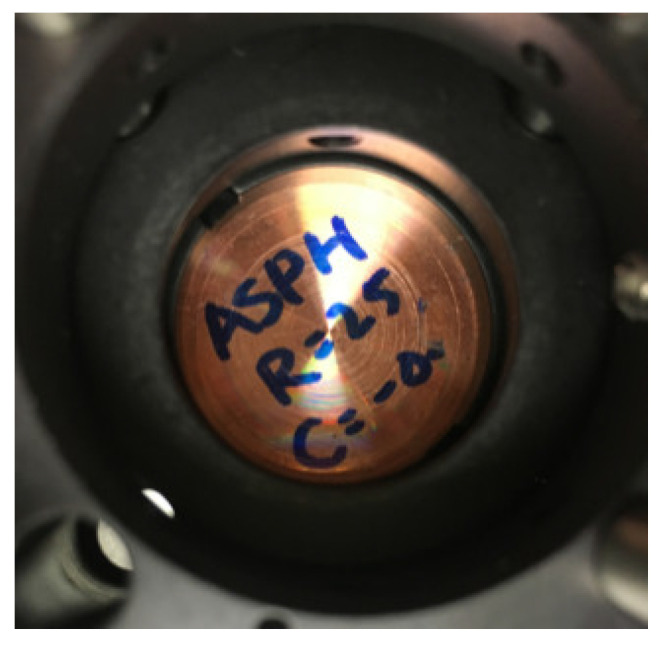
The measured aspherical mirror.

The optical path of measuring the plan mirror is simple. Therefore, we talk about the second condition, where there is a lens (NA of 0.25 and radius less than 10 mm, which can be used to measure the full radius of tested mirror) in R1. After the light (L1) gets into the tested mirror, as shown in Figure 7, the light (L2) with the information of the tested mirrors reflect and go through the Pellicle beam splitter; L2 is divided two parts: one part can be neglected and the other part gets into R2, called L3. After all of them are coaxial, we began to capture the pictures with CCD (on an electrical movable stage that can be controlled to ensure accurate focus and defocus).

Third: Adjust the len2 location around the focus location, and use the movable stage to obtain some defocused and focused images.

Fourth: Dispose of the collected images with the improved PR algorithm; obtain the aberration of the measured aspherical mirror.

**Figure 8:**
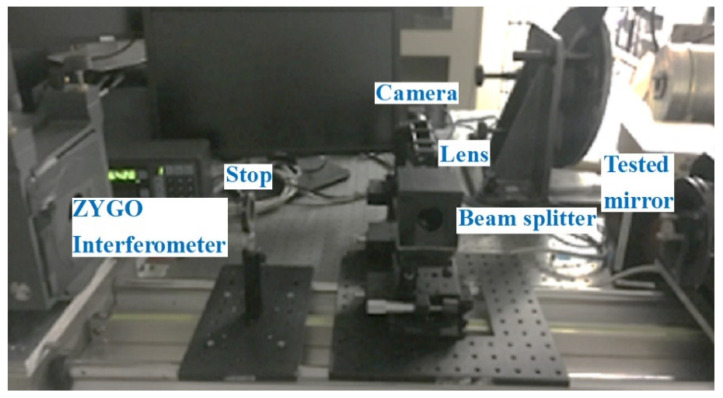
The measurement experiment with the ZYGO interferometer.

Fifth: Use the ZYGO interferometer to measure the aspherical mirror. The estimation experiment with the ZYGO interferometer is shown in Figure 8.

### 3.3. Experimental Results and Discussion

**Figure 9:**
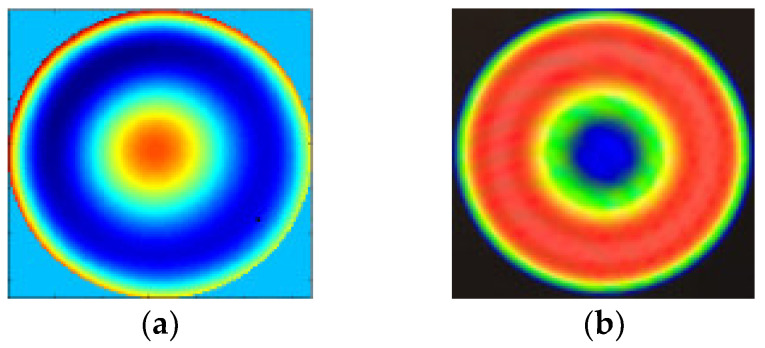
The measurement results with (**a**) PR: RMS = 0.674λ, PV = 3.12λ; (**b**) ZYGO interferometer: RMS = 0.632λ, PV = 2.155λ.

We disposed the collected seven images with the improved PR algorithm and obtained the measured results of the aspherical mirror, as shown in Figure 9a. The measured result with the ZYGO interferometer is shown in Figure 9b. It can be seen from the two results from Figure 9a,b that there was an agreement among the error distribution and the RMS value of the ZYGO interferometer. The PV of two results was a little different from the RMS value. There are several reasons for the difference in PV. First, there was a smoothing process when using the interferometer for surface detection, and Figure 9b was obtained after matting (removing boundary Burr). Second, in the process of calculation, we calculated the points in the whole mask circular area. Some values of the PR method may deviate from the true value more at the boundary, corresponding to Figure 9a, which was obviously different from the edge area. Therefore, although the RMS of the whole mask could not be greatly affected, it will be greatly different from PV.

It can be seen from the above experimental results that the PR method had the feasibility and accuracy in measuring the aspherical mirror and the improved PR method could achieve the considerable accuracy as ZYGO interferometer, which showed that the PR method could meet the needs of practical engineering and lay the foundation for the measurement of free-form surfaces with our measurement technology in the future. Furthermore, the PR method has the advantages that a ZYGO interferometer does not have:

(1) The impact of platform vibration on the PR system is small, even negligible;
(2) PR system has a simple structure, and can even detect the whole optical system in place by using the existing camera on the imaging system without any change to the optical path; and
(3) Better measurement accuracy can be obtained with fewer sampling points by CCD in the PR system.

## 4. Conclusions

This paper set up an experimental arrangement with the method of PR technology based on the improved PR algorithm to measure the aspherical optical surfaces. During the construction of the experiment, we explained in detail the installation and adjustment methods for high-precision measurements. From the compared measurement results of the aspherical mirror with PR and a ZYGO interferometer, we obtained an agreement between the error distribution, the PV value, and the RMS value of the ZYGO interferometer, which demonstrates the feasibility and effectiveness of the PR technology in estimating the wave-front and aberrations of the aspherical optical surfaces. Furthermore, the results of the experiment further verify that PR technology is a viable and realistic method in our later research in free-form surface measurement.
